# Availability of medicines in public sector health facilities of two North Indian States

**DOI:** 10.1186/s40360-015-0043-8

**Published:** 2015-12-23

**Authors:** Shankar Prinja, Pankaj Bahuguna, Jaya Prasad Tripathy, Rajesh Kumar

**Affiliations:** School of Public Health, Post Graduate Institute of Medical Education and Research, Chandigarh, 160012 India

**Keywords:** Essential medicines, Availability, Public sector, Generic, Anti-hypertensive, Procurement, Inventory management, Universal health care

## Abstract

**Background:**

Access to free essential medicines is a critical component of universal health coverage. However availability of essential medicines is poor in India with more than two-third of the people having limited or no access. This has pushed up private out-of-pocket expenditure due to medicines. The states of Punjab and Haryana are in the process of institutionalizing drug procurement models to provide uninterrupted access to essential medicines free of cost in all public hospitals and health centres. We undertook this study to assess the availability of medicines in public sector health facilities in the 2 states. Secondly, we also ascertained the quality of storage and inventory management systems in health facilities.

**Methods:**

The present study was carried out in 80 public health facilities across 12 districts in Haryana and Punjab states. Overall, within each state 1 MC, 6 DHs, 11 CHCs and 22 PHCs were selected for the study. Drug procurement mechanisms in both the states were studied through document reviews and in-depth interviews with key stakeholders. Stock registers were reviewed to collect data on availability of a basket of essential medicines −92 at Primary Health Centre (PHC) level, 132 at Community Health Centre (CHC) level and 160 at tertiary care (District Hospital/Medical College) level. These essential medicines were selected based on the Essential Medicine List (EML) of the Department of Health (DOH).

**Results:**

Overall availability of medicines was 45.2 % and 51.1 % in Punjab and Haryana respectively. Availability of anti-hypertensives was around 60 % in both the states whereas for anti-diabetics it was 44 % and 47 % in Punjab and Haryana respectively. Atleast one drug in each of the categories including analgesic/antipyretic, anti-helminthic, anti-spasmodic, anti-emetic, anti-hypertensive and uterotonics were nearly universally available in public sector facilities. On the contrary, medicines such as thrombolytics, anti-cancer and endocrine medicines were available in less than 30 % in public sector facilities. Among the medicines which were not available at the time of survey in Haryana, nearly 60 % of them were out of stock for 3–6 months whereas 8 % of them were out of stock for more than 6 months.

**Conclusion:**

Health system needs to be strengthened by making essential medicines available for patients. Ensuring access to free medicines is likely to reduce private expenditure on medicines, which is a long-term, sustainable way to towards universal health coverage in India.

**Electronic supplementary material:**

The online version of this article (doi:10.1186/s40360-015-0043-8) contains supplementary material, which is available to authorized users.

## Background

The provision of affordable, high quality and appropriate essential medicines is a vital component of a well-functioning health system. Nearly 10 million lives could be saved by improved access to essential medicines, of which 4 million are in Africa and South-East Asia alone [1]. However, providing universal access to essential medicines is a major challenge in low and middle income countries (LMICs) [2]. Although there is reasonably sufficient information from the developed nations regarding access to essential medicines, the data from LMICs is often weak and fragmented.^1^Recent studies report that in LMICs the average availability of medicines in the public sector is only 35 % [3]. According to a World Health Organization (WHO) report, almost 68 % of the people in India have limited or no access to essential medicines [2]. Poor availability of medicines in the public sector has pushed up household out-of-pocket (OOP) expenditure, making them the largest household expenditure item after food [4]. Up to 90 % of the population in developing countries purchase medicines through OOP payments [5]. Nearly 80 per cent of India’s health care expenditure is borne by patients OOP, of which medicines constitute 70 % [6]. Another study in the three North Indian states of Haryana, Punjab and Chandigarh also reported that medicines constituted 19-47 % of hospitalization expenditure and 59 to 86 per cent out-patient department (OPD) expenditure borne out-of-pocket by households in public sector [7].

Access to free of cost essential medicines is a critical component of universal health coverage. This is being considered as a key intervention in Government of India’s (GOI) proposed National Health Assurance Mission (NHAM) in 2014 [8]. Ensuring availability of free essential medicines significantly reduces the burden on private OOP expenditures. Moreover, it provides financial risk protection to population, most vulnerable to pay catastrophic health expenditures [9]. Every Indian state follow an independent mechanism of procurement of medicines. Various drug procurement models have been implemented in states like Tamil Nadu, Kerala and Rajasthan towards achieving the goals of universal health coverage. This has resulted in lower price and better availability of medicines through efficient supply chain management [10, 11]. Tamil Nadu follows a mixed procurement system (80 % centralized and 20 % decentralized) whereas in Kerala it is completely centralized, for acquiring medicines. Moreover, the procurement systems in both the states are completely autonomous with minimal interference of state government. The governance structures created in the two states (Punjab and Haryana) are also very similar to the models followed in Kerala and Tamil Nadu. This has also led to similar processes being followed for inviting the suppliers, pricing, selection of essential medicines, setting up of distribution channels etc. [10]. Other State Governments in India are planning to replicate such models to provide essential medicines free of cost at all public health facilities.

The current study is a comprehensive effort to assess the availability of medicines in public sector health facilities in two North Indian states of Punjab and Haryana. Both the states have recently instituted procurement mechanisms similar to states like Tamil Nadu and Kerala to provide uninterrupted access to essential medicines of good quality and free of cost in all government institutions [10, 12]. This study reflects the baseline situation on availability of medicines prior to setting up of new procurement systems. This evidence not only reflects on the baseline situation but will also serve as a reference to evaluate the impact of new systems of procurement and their cost effectiveness. In this paper, we report the availability of essential medicines at various levels in public health facilities in Haryana and Punjab. Additionally, we also assessed the storage and inventory systems of medicines in the public health facilities. As a part of large study, the system of procurement, pricing and distribution was also reviewed.

## Methods

### Study setting and sampling

The state of Haryana is one of the wealthier states of India with the third highest per capita income in the country in the year 2012–13 [13]. Nearly two-thirds of the 25 million population of the state resides in rural areas [14]. Punjab is another prosperous agricultural state with a population of 28 million with similar proportion belonging to rural areas [15]. As in rest of India, a 3 tier health care service delivery system caters to needs of population in Haryana and Punjab. The primary level includes Sub-centres (SCs) and Primary Health Centres (PHCs). The Community Health Centres (CHCs) and District Hospitals make up the secondary level, and the Medical Colleges are at the tertiary level. The Sub-centre (SC) is the most peripheral health institution for every 5000 population. SC is staffed by an Auxiliary Nurse Midwife (ANM). Primary Health Centres (PHCs) which are manned by medical officer (doctor) provide primary care to a population of around 30000.Community Health Centres (CHCs) provide secondary care services to a population of around 1, 20,000. District Hospitals (DHs) provide specialist secondary care facilities at the district level whereas Medical colleges (MCs) provide tertiary care services.

A multi-stage stratified random sampling was followed for district selection. In the first stage, all districts were stratified in three categories based on the human development score, i.e. high, medium and low status of development [16]. Secondly, 2 districts were selected randomly from each strata. The selected set of districts also ensured a geographical representation of the state.

In second stage, a total of 80 public health facilities were chosen for the study so as to cover all levels of health care delivery system, i.e. primary, secondary and the tertiary. The study sample included 1 medical college from each state and 1 district hospital (DH) in each district. We selected almost 30 % of CHCs in each district and two PHCs under each CHC were randomly selected. Overall, within each state1 MC, 6 DHs, 11 CHCs and 22 PHCs were selected for the study. The final sample thus comprised of 2 tertiary care medical colleges, 12 district hospitals, 22 CHCs and 44 PHCs for the two states. The details regarding the sampled public health facilities are given in Table 1.

**Table 1:** Summary of the sample of public health facilities selected

Characteristics	Total	Sample selected	% of Total
**Districts**	43	12	27.9
**District Hospital (1 from each selected district)**	43	12	27.9
**CHCs (30 % of CHCs in the selected districts)**	241	22	9.13
**PHCs (2 from each CHC)**	896	44	4.91
**Medical College (1 from each state)**	6	2	33.3
**Total Public Facilities Sampled**	1186	80	6.75

***Note:*** *Source: For information on total number of public health facilities - Rural Health Statistics 2012, Ministry of Health and Family Welfare, Government of India – Accessed on 18/05/2014,* PHC = Primary Health Centre, CHC = Community Health Centre

### Data collection

Primary data collection was undertaken to meet the objective of the study in the period of June to July month of year 2013. Primary data included assessment of availability of medicines, storage, inventory management and stock-outs at facility at different level of facilities in sample districts of 2 states.

For assessing availability and stock-outs of essential medicines at the facility, the ‘Facility Level Medicine Availability and Stock-out Tool’ was used (Additional file 1: Table S1). The tool was used to collect data on drug availability on the day of the survey, medicine stock-out position for the previous six months from the date of the survey and the duration of stock-outs. A team of trained investigators visited pharmacy outlets at public health facilities with survey tools to capture availability and stock-outs of medicines. The investigators were post-graduates with previous experience of social sciences research in health system. Moreover, one member of the team who collected the data was a medical officer. A one week training was undertaken for field investigators to train them on data collection methods, tools, familiarization of drug procurement and management at facility level. They carried out structured interviews with the store-in-charge/medical officer/pharmacist/any other person handling procurement and dispensing at the facility level. Data was extracted from stock registers available in each facility. A list of medicines to be surveyed was prepared after reviewing the National List of Essential medicines (NLEM), state Essential Medicine List (EML), and medicines provided under National Health Programs. Availability of medicines were assessed at primary, secondary and tertiary health facilities against the basket of 92, 132 and 160 medicines respectively selected according to therapeutic categories. In addition to the medicines under primary care facilities, secondary care facilities had 40 other medicines, whereas, in addition to the medicines under the primary and secondary care facilities, tertiary care facilities had 28 other medicines belonging to categories such as anti-cancer medicines, hormonal supplements, certain antibiotics like cefixime, vancomycin and other medicines like allopurinol, urokinase, glucagon, lithium carbonate etc. The list of medicines has been given as an appendix. A structured tool was used to collect data on stock-out and availability of survey medicines in public health facilities. Ten percent of medicines available as per records were randomly cross-checked in the store. For the medicines not available on the day of the survey, the number of days of stock outs in last 6 months was recorded by manual checking of stock registers.

Additionally as a part of overall study, data on structures of procurement system, distribution and pricing was collected, although, in this paper we limited our focus only on availability of medicines in two states.

### Data analysis

Data was entered in Microsoft Access 2010, and analysed using SPSS version 21.Two types of analyses were done to assess the availability of medicines. In first analysis, availability of all the medicines under a particular therapeutic category was assessed, whereas in second analysis, even a single drug (out of total medicines under each therapeutic category) available under a therapeutic category was considered as availability of that particular therapeutic category. Both the type of analysis are explained below:

## Availability of medicines by therapeutic category

The medicines were classified into therapeutic categories. A drug was considered available if it was in stock on the day of the survey. Availability of a therapeutic category in a facility is explained by the formula: (**n**/**N**) * **100**, where n is the number of medicines available within that category in a facility on the day of the survey and N is the total number of medicines within that category that should be available as per the list of medicines prepared. For a particular level of facility (say for example PHC level in Punjab), overall availability of a particular category of medicine is given by the formula:

$$ \frac{{\displaystyle \sum {\left({\mathbf{n}}_{\mathbf{i}}\right)}^{*}\mathbf{100}}}{{\mathbf{M}}^{*}\mathbf{N}} $$

Where, n_i_ is the number of medicines available within a therapeutic category in a particular facility and M is the number of facilities in that particular level of care (in this case, 22 PHCs were surveyed in Punjab) and N is the total number of medicines within that category that should be available as per the list of medicines being surveyed. Thus N*M gives the total number of medicines in that category that were surveyed in all PHCs in Punjab. As an example, if 6 (N) antihypertensive medicines were evaluated for availability in 22 PHCs (M) of Punjab, then a total of 132 items (M*N) are being evaluated. Against a set of these items, if 4 antihypertensive drugs are available in 12 facilities and 3 antihypertensive medicines are available in remaining 10 facilities, then the overall anti-hypertensive medicine availability at PHC level in Punjab is 59 % [((4*12) + (3*10))*100/132].

Overall availability of a particular category of medicine across all levels of care in a state is given by the formula:

$$ \frac{{\displaystyle \sum \left({\mathbf{n}}_{\mathbf{i}}\right)\ast \mathbf{100}}}{{\displaystyle \sum {\mathbf{M}}_{\mathbf{i}}\ast \mathbf{N}\mathbf{i}}} $$

i ranges from 1–4, where 1,2,3,4 stands for four levels of care namely PHC, CHC, DH and MC.

Where, n_i_ is the number of medicines available within a therapeutic category in a particular facility and M_i_ is the number of facilities in that particular level of care and N_i_ is the total number of medicines within that category that should be available as per the basket of medicines in that particular level of care.

Each dosage form of medicine was considered as a separate entity in the basket of medicines. This was in concordance with the way it is required as per the Essential Drug List in India. It is also justified theoretically, as each of these medicines in the specific dosage form should be available for dispensing at the health facility. In another analysis, a particular category of drug was considered available when at least one drug from the category was available. Average duration of stock out was computed for those medicines which were not available on the day of survey. We categorized stock out duration as less than 1 month, 1–3 months, 3–6 months and more than 6 months.

### Ethical approval

This study was approved by ethics committee of Post Graduate Institute of Medical Education and Research (PGIMER), Chandigarh (India). Approval was obtained from concerned authorities of health departments in both the states. Prior to data collection, administrative approval for carrying out the study was taken from the Civil Surgeon (head of health administration) at the district level in all 12 districts. Written informed consent of the chief pharmacist and medical officer (MO) was sought prior to the interviews and review of records. This study was funded by the Public Health Foundation of India (PHFI), New Delhi.

## Results

### Storage and inventory management system of medicines

Around 95 % of public health facilities in Punjab had the dedicated storage space with temperature control and proper ventilation. Cold storage facility was available in 85 % of facilities in Punjab. All the public health facilities in Haryana had the dedicated space for storage along with cold storage facility for medicines. Around 89 % had temperature control mechanism and proper ventilation in Haryana. Medicines were stored directly on floor in Punjab and Haryana in 28 % and 18 % of public health facilities respectively. Evidence of pests in drug stores were found in 10 % of facilities in Haryana whereas it was only 2 % for Punjab.

All the public health facilities (40) in Punjab and 37 (93 %) health facilities in Haryana were found to maintain scientific inventory management method of First Expiry First Out (FEFO) (Table 2). At the PHC level, the average interval of indenting the medicines was 96 days in Punjab, whereas it was only 37 days in Haryana. At the district level, the average indenting interval was 26 and 45 days in Punjab and Haryana respectively. At the PHC level in Punjab, it took more than three weeks (25 days) for the medicines to reach the facility after indenting, whereas in Haryana it took only 7 days. At the CHC level it took around 2 weeks for the medicines to reach the facility in both the states. The average duration to receive medicines at district hospital was 17 days and 49 days in Punjab and Haryana respectively (Table 2).

**Table 2:** Inventory Management Process in Public Health Facilities of two North Indian States

Inventory management process	Punjab				Haryana			
	PHC n = 22	CHC n = 11	DH n = 6	MC n = 1	PHC n = 22	CHC n = 11	DH n = 6	MC n = 1
Methods of Inventory management (FEFO)	22	11	6	1	20	11	5	1
Methods of Inventory management (FIFO)	0	0	0	0	2	0	1	0
Average interval of indenting (days)	96	39	26	NR	37	42	45	NR
Average numbers of medicines indented	50	115	99	NR	63	69	166	NR
Average number of medicines received per indent	71	90	NR	NR	79	84	50	NR
Average number of days to receive medicines	25	16	17	NR	7	15	49	NR

PHC = Primary Health Centre; CHC = Community Health Centre; DH = District Hospital; MC = Medical College; NR = Not recorded; FEFO = First-Expiry-First-Out; FIFO = First-In-First-Out

### Availability of essential medicines at public health facilities by therapeutic category

Overall availability of medicines in Punjab was 45.2 % which varied from 48 % at the DH and PHC to 44 % at CHC level (Table 3). The availability of medicines at a MC was only 4.4 %. In Punjab, almost 70 % of public health facilities had the medicines in categories of ant-helminthic/anti-parasitic, antispasmodic, antiemetic and uterotonics available at the time of survey. However, anti-cancer, thrombolytics and endocrine related medicines were available in less than 10 % of facilities. At the PHC level, the availability of anti-hypertensives was 60%whereas only around one-third of PHCs had anti-diabetics, antidepressants/antipsychotics and anti-asthmatics available on day of survey. At the DH, availability of anti-diabetics, NSAID, anti-allergic and anti-hypertensives was more than60%. In the medical college in Punjab, apart from some anti-bacterials, anti-cancer agents and anaesthetic medicines other essential medicines were found out of stock (Table 3).We also assessed the availability of medicines considering if any of the drug falling under a therapeutic category is available at the time of survey. Only 5 % facilities had a single drug in anti-cancer category whereas any thrombolytic, endocrine, anti-fungal and anti-viral drug was available in 23 %, 25 %, 30 % and 35 % of public health facilities respectively (Table 4).

**Table 3:** Availability of Medicines (%) by Therapeutic Category in Public Health Facilities

Drug category	Punjab					Haryana				
	PHC	CHC	DH	MC	Total	PHC	CHC	DH	MC	Total
Analgesic/Anti-Pyretic/NSAID	74.3	49.1	61.1	0	58.7	70.0	43.6	47.2	33.3	53.6
Anti-bacterial	50.8	52.5	54.2	12.5	50.7	59.1	59.8	59.7	62.5	59.6
Anti-allergic	49.6	58.3	63.9	8.3	53.4	59.7	62.0	66.2	75.0	61.8
Vitamins & Minerals	53.7	46.5	50.0	0	49.2	60.4	60.6	68.5	44.4	61.4
Anti-asthmatic	31.0	36.4	47.2	0	34.2	50.0	54.5	72.2	50.0	54.6
Antacid	42.9	41.2	40.5	0	40.0	71.2	66.7	69.0	71.4	69.1
Anti-helminthic/Anti-parasitic	90.5	68.2	70.8	0	77.0	54.5	72.7	66.7	75.0	63.0
Anti-fungal	0.0	31.8	50.0	0	36.1	0.0	50.0	41.7	50.0	47.2
Anti-spasmodic	71.4	68.2	75.0	0	69.2	63.6	63.6	75.0	0.0	63.8
Anti-emetic	90.5	90.9	100.0	0	89.7	77.3	77.3	66.7	100.0	76.3
ORS	61.9	54.5	83.3	0	61.5	100.0	100.0	100.0	100.0	100.0
Anti-hypertensive	60.5	61.4	61.7	0	59.1	59.7	58.6	55.0	60.0	58.5
Anti-diabetic	35.2	54.5	63.3	0	44.1	38.2	49.1	76.7	40.0	47.0
Thrombolytic	0.0	4.3	20.0	0	9.9	0.0	10.2	46.7	50.0	26.6
Antidepressant/Anti-psychotic/Antiepileptic	31.9	30.1	41.7	0	32.2	27.7	34.1	47.6	64.3	34.4
Anti-viral	0.0	29.2	33.3	0	28.9	0.0	13.6	27.8	0.0	18.6
Uterotonics	88.1	83.8	63.3	0	76.3	68.2	66.7	66.7	80.0	67.9
Other endocrine medicines	9.5	2.4	4.8	0	5.9	13.6	21.2	28.6	14.3	19.6
Miscellaneous	34.9	34.5	38.2	5.9	34.4	35.4	39.4	37.3	23.5	36.5
Anti-cancer medicines	0.0	0.0	4.2	25	4.4	0.0	9.1	4.2	25.0	7.7
Anaesthetic agents	28.6	34.1	45.8	25	32.7	27.3	29.5	66.7	50.0	34.4
Total	48.1	44.0	47.8	4.4	45.2	50.9	49.3	54.1	50.6	51.1

***Note:*** Figures given in the table are percentages. Overall, 2 MC, 12 DH, 22 CHC and 44 PHC were covered in this study in the two states Punjab and Haryana. Where; PHC = Primary Health Centre, CHC = Community Health Centre, DH = District Hospital, MC = Medical College

**Table 4:** Availability of Medicines (any one drug available within the therapeutic Category) by Therapeutic Category at different level of public health facilities

Drug category	Punjab					Haryana				
	PHC	CHC	DH	MC	Total	PHC	CHC	DH	MC	Total
Analgesic/Anti-Pyretic/NSAID	100	100	100	0	98	100	100	100	100	100
Anti-bacterial	100	100	100	100	100	100	100	100	100	100
Anti-allergic	100	100	100	100	100	100	100	100	100	100
Vitamins& Minerals	100	100	100	0	98	100	100	100	100	100
Anti-asthmatic	95	100	100	0	95	86	100	100	100	93
Antacid	95	100	100	0	95	91	100	100	100	95
Anti-helminthic/Anti-parasitic	95	100	100	0	95	95	100	100	100	98
Anti-fungal	0	64	83	0	30	0	100	83	100	43
Anti-spasmodic	91	82	100	0	88	91	100	100	0	93
Anti-emetic	95	91	100	0	93	91	91	83	100	90
ORS	64	64	83	0	65	100	100	100	100	100
Anti-hypertensive	100	100	100	0	98	100	100	100	100	100
Anti-diabetic	100	100	100	0	98	91	100	100	100	95
Thrombolytic	5	27	83	0	23	0	55	100	100	33
Anti-depressants/Antipsychotics	100	100	100	0	98	100	100	100	100	100
Anti-viral	0	73	100	0	35	0	27	83	0	20
Uterotonics	91	100	83	0	90	86	100	100	100	93
Endocrine medicines	32	18	17	0	25	32	55	83	100	48
Miscellaneous	100	100	100	100	100	100	100	100	100	100
Anti-cancer medicines	0	0	17	100	5	0	9	17	100	8
Anaesthetic agents	95	100	100	100	98	73	82	100	100	80
Total	74	82	89	24	77	73	87	93	90	80

***Note:*** Figures given in the table are percentages. Overall, 2 MC, 12 DH, 22 CHC and 44 PHC were covered in this study in the two states Punjab and Haryana. Where; PHC = Primary Health Centre, CHC = Community Health Centre, DH = District Hospital, MC = Medical College

In Haryana, the overall availability of medicines was 51.1 % with highest at DH level (54.1 %) followed by PHC (50.9 %), MC (50.6 %) and CHC (49.3 %). In Haryana, overall drug availability in public health facilities was good (almost 70 %) in categories like antiemetic, antacid and uterotonics whereas poor (less than 30 %) in therapeutic categories such as anticancer, antiviral, endocrine and thrombolytics. Less than 40 % of the PHCs had anti-diabetics and anti-depressant/anti-epileptic available on the day of survey. Availability of anti-asthmatics, anti-allergics, antacids, anti-diabetics, anti-spasmodic and anti-emetics was more than two-third at the DH level (Table 3). Among the anti-bacterials, anti-diabetics, anti-hypertensives, anti-asthmatics, anti-depressants/anti-psychotics, vitamins and antacids, at least one drug from each category was present in almost all the public health facilities. However, not even a single drug among therapeutic categories like anti-cancer, anti-viral, thrombolytics and endocrine medicines was present in around 95 %, 65 %, 77 % and 75 % of health facilities of Punjab respectively and; 92 %, 80 %, 67 % and 52 % of health facilities of Haryana respectively (Table 4).

### Stock-outs of essential medicines

**Fig. 1:**
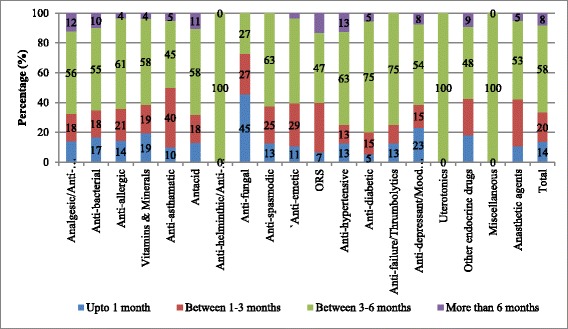
Duration of Stock out (For medicines not available at the time of survey) in public health facilities, Haryana

Among the medicines which were not available at the time of survey in Haryana, nearly 60 % of them were out of stock for 3–6 months whereas 8 % of them were out of stock for more than 6 months (Fig. 1). Nearly 60 % of analgesics, anti-allergics, anti-spasmodics, anti-hypertensives, antacids and vitamins, which were not available at the time of survey, were out of stock for the last 3–6 months. Among the anti-diabetics not available, 75 % were out of stock for 3–6 months (Fig. 1).Average number of days of stock out for analgesics, anti-bacterial, anti-helminthic, anti-fungal, anti-diabetic and uterotonics was 160–180 days.

**Fig. 2:**
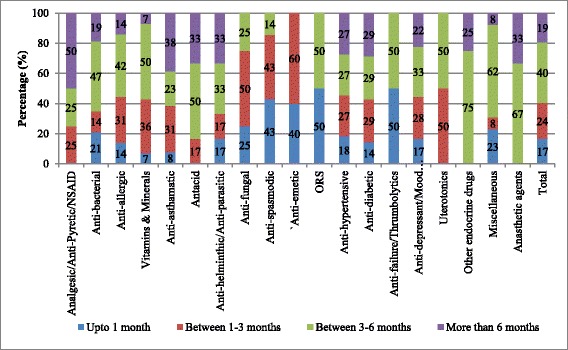
Duration of Stock out (For medicines not available at the time of survey) in public health facilities, Punjab

In Punjab, among the medicines which were not available at the time of survey, nearly 40 % of them were out of stock for 3–6 months whereas 19 % of them were out of stock for more than 6 months. About 27 % of anti-hypertensives and 19 % of anti-diabetics were found to be out of stock for more than 6 months (Fig. 2). Average number of days of stock out for analgesics, endocrine, anti-asthmatic and anti-helminthic medicines was 231 days, 211 days, 193 days and 186 days respectively.

### Drug procurement models in Haryana and Punjab

In Haryana, there are three main sources of funding for the procurement of essential medicines. Firstly, the State Government provides funds in its budget for purchase of medicines. Secondly a grant-in-aid received from the Government of India as part of its flagship program National Health Mission (NHM) comprises of a budget-line for medicines. Finally, the untied funds available with the health facility committee can be used for purchasing medicines with a minor contribution comes from the money collected as a part of user charges. In decentralized system under National Health Mission (NHM), untied funds are given to health facilities which can be used to improve the quality of care. One of these activities on which untied funds can be used is purchase of essential medicines. In 2011–12, total budget for medicines in Haryana was INR 350 million, out of which the contribution from NHM and State Government is 43 % and 57 % respectively. The procurement and distribution model for medicines followed in Haryana was set up in 2009. In this system, procurement of medicines was decentralized at district level. A task committee was formulated at state level comprising of experts including pharmacologists, state drug controller and other health system program managers who reviewed the State EML every year.

In this procurement system, funds were disposed to *District Health Societies* for the procurement of medicines & consumables. As per the procurement policy, all medicines and medical consumables were to be purchased under Pharmacopoeia generic names. Medicines listed in the EML, were called through open tendering at district level. Open tendering system is a process in which quotations are invited from potential manufactures or suppliers (mainly pharmaceutical firms) by authorities (generally Director Supplies and Disposal) through a proper channel. Only firm (i.e. Pharmaceutical manufacturers and suppliers) which had Good Manufacturing Practices (GMP) Certificate in accordance with the WHO recommendations issued by Central / State Drug Control Authorities were eligible. Two or three firms (L1, L2, L3) were approved for each drug with a validity of two years. Random samples of medicines from each consignment of medicines were tested at government approved laboratories. Sample which did not meet quality standards were rejected entirely and costs were recovered from the firm. A proper channel was followed between health facilities and *District Health Societies* for distribution of procured medicines. The frequency of issuing indents for obtaining medicines from district store was used for assessment, forwarded by Chief Pharmacist. Distribution of medicines was done to all level of facilities after assessing the indent against previous demand and utilization pattern. Every health facility has been issued a passbook, in which all the records related to demands through indents, stock-out and utilization status of medicines are maintained. When a facility raise its demand through indent, these pass books are analyzed and serve as a useful tool for assessments. ‘Demand’ for medicines implies a manifestation of medicine requirement in the form of an ‘indent’ which a health facility puts up to the local warehouse or supplier of medicine, for provision of the same.

Similar to Haryana, Punjab also had decentralized procurement system. For procurement of medicines in Punjab the districts placed their orders to State. Medicine budget was released from state to district dependent upon demand raised by district. Majority of the procurement was done at district level utilizing these funds except few medicines which were directly procured by State. All the facilities retained the revenue generated in the form of user charges. Almost 40-45 % of revenue generated at facility level against user fee charges was also utilized for medicine purchase. Only emergency medicines which are not available at state/district level stores or not approved to be part of EML, can be purchased through this route under the name of ‘local purchase’. Almost every district had their dedicated drug stores. The tenders of medicines were floated at district level. Medicines are purchased in bulk at state level. This is a paradigm shift from the past, where medicine procurement happened at the district level. For medicines which could not be purchased through tendering process due to reasons like non-participation in bidding from firms, the state empanelled chemist shops from which health facilities could purchase medicines directly at fixed prices. In such cases, medicines are purchased from chemists with some discount (from their profit margin) on market price.Adherence to procurement guidelines was poor in the decentralized system. Alike Haryana, indenting of medicines at facilities was done through pharmacists and supply obtained from district level.

## Discussion

Availability of free essential medicines is critical to deliver universal health care. Lack of access to medicines causes households to face financial catastrophe through increased OOP expenditure [17]. Health spending in India was 4 % of Gross Domestic Product (GDP) in the year 2012 with public share being one-third only [6]. Out-of-pocket expenditure persists to be major source of health spending out of which almost 70 % of the OOP burden is caused due to medicine expenses [6, 7, 18]. OOP on medicines alone pushes 2.2 % of the population below the poverty line annually [19]. Unavailability of medicines is also the major reason for dissatisfaction among patients [20]. Besides that, lack of supplies impacts upon staff morale through community pressure [21]. We found that the overall availability of essential medicines in public sector health facilities and hospitals was 45.2 % and 51.1 % in Punjab and Haryana respectively which is well below the WHO standards of 80 %. Similar levels of drug availability were also reported from studies in other low-middle-income countries [3, 22, 23].

Recent surveys in India show significant variation in the availability of essential medicines in different states. The mean availability of a selected basket of essential medicines in Bihar was 43 % as compared to 88 % in Tamil Nadu [24]^.^ Another study in Delhi reported mean availability to be 41 % and 23 % in facilities under State government and Municipal Corporation respectively [25]. Surveys carried out in six states in India using a standard WHO methodology reported poor availability of medicines in the public sector with median availability ranging from 0–30 % [26]. Analysis of National Sample Surveys showed that during the mid-1980s one-third of medicines during hospitalization were supplied free of cost which declined to 9 % during 2004. In case of outpatient care, free medicine supply declined from 18 % to about 5 % over the same period [27]. Low availability of essential medicines at public health facilities force patients to purchase medicines from private pharmacies where there is higher availability of medicines and for many medicines, only one brand of the product is available usually the costly one [25]. Therefore, the patients have no choice but to buy that particular costly branded product thereby incurring catastrophic drug expenditure.

We found that most of the facilities followed a scientific method of inventory management (FEFO). However, the average number of days needed to receive the medicines varied from 4 to 14 weeks in a public sector facility which might explain frequent stock-outs and thus pointing to the inefficiencies in the procurement/distribution system. Low availability of medicines in the public sector and frequent stock-outs has also been reported in other studies due to factors such as under-funding, inaccurate forecasting, inefficient procurement/distribution mechanism in the supply chain, prescription practices leading to prescriptions for medicines outside the public health system and the notion that medicines supplied through the public system are of low quality [3].

Non-communicable diseases require long-term compliance to treatment sometimes even for a lifetime. With the rising burden of non-communicable diseases, poor availability of anti-hypertensives, anti-diabetics, anti-asthmatics and anti-depressants/anti-psychotics, as reported in the present study, force patients to purchase medicines from the private sector or forego treatment if they cannot afford it. Another study also reported poor availability of essential medicines for chronic diseases in six LMICs in public sector but better availability in the private sector [28].

Various recommendations to improve availability of essential medicines in the public sector are proposed in the literature such as – increase the budget for medicines; formulate standard treatment guidelines (STGs) and EML based on STGs; separate EML for primary care and other levels of care; procurement and distribution of medicines based on EML; procurement by generic name; efficient transparent and accountable procurement and distribution system; use of robust IT systems; utilizing local supply options; supportive legislation and regulation; better prescription practices; and regular monitoring and evaluation of the system [28–30].

Different procurement models centralized, decentralized and mixed have been tried in India. A review of literature reports that autonomous centralized procurement organizations such as in Tamil Nadu and Kerala were more efficient in relation to payments to suppliers, had relatively lower drug procurement prices and managed the inventory more scientifically [10]. Many states are now trying to replicate the Tamil Nadu Medical Services Corporation (TNMSC) model of centralized tendering and purchase of medicines. However, critical success factors of each model need to be carefully analysed to see if they are valid in another state context before replicating them. The key factor for the success of TNMSC is its autonomy coupled with able leadership. Besides, for centralized procurement models it is necessary to have optimum number of warehouses, adequate transportation facilities to transfer supplies from warehouses to user facilities, a robust IT system for real-time monitoring of stocks and online ordering and dispensing, which requires considerable amount of capital expenditure. Despite implementing similar reforms, the state of Odisha did not garner similar level of success as in Tamil Nadu. With poor autonomy of the drug procurement agency, Odisha grappled with implicit state level problems of poor governance, lack of political will and ineffective leadership. Thus, Odisha, with poor infrastructure, insufficient investment and inherent system related problems has failed to reap the benefits of a centralized pooled procurement model [10]. There are some successful models as well. The pooled procurement system introduced in Delhi along with carefully selected essential medicine list, standard treatment guidelines and focus on rational prescribing has resulted in uninterrupted supply of good quality medicines and has brought down the procurement costs of medicines saving nearly 30 % of the annual medicines bill which were mobilized for procuring more medicines. This in turn improved availability of medicines (more than 80 %) at health facilities [31].

We acknowledge that evaluating availability of basket of medicines at overall level or by the therapeutic category, between and within sectors, or between the states may not be the most relevant. In order to circumvent this problem, we have revised our analysis (Additional file 2: Table S2), in terms of availability of each and every medicine within each therapeutic category. Nonetheless, assessing the availability for the pool “basket of medicines” is also relevant from policy perspective as all the medicines which are part of the surveyed basket of medicines, constitute part of the essential drug list in India. As a result, none of the medicines being surveyed are non-essential or non-recommended.

Governance in procurement process plays a critical element for optimum utilization of resources in public health system, given the technical complexity of drug procurement. A transparent, efficient and cost-effective procurement process is desirable. The procurement organizations in Tamil Nadu, Rajasthan and Kerala are autonomous. The idea of having an autonomous corporation for procurement and distribution of medicines is to enable it to function more transparently by avoiding the plausible procedural delays and make quick decisions for better functioning of the organization. It also has an advantage of economies of scale wherein there is better negotiation with the suppliers. States such as Uttar Pradesh, Bihar, Gujarat and many others have Central Medical Stores Department under Department of Health and Family Welfare responsible for procurement of medicines and other medical supplies. But the role of this department is limited up to finalization of rate contract with suppliers. Actual procurement is carried out at district levels by Chief Medical Officers at district level or through head of health facilities at district and sub-district level.

Punjab and Haryana have procurement cells that are a part of the state health services which may have a bearing on the efficiency of the processes. They are in a transition to adopt centralized procurement and decentralized distribution model. In Punjab, a robust IT system needs to be integrated into the procurement mechanism to enable tendering process and real-time inventory control. Although both the states have mandated prescription by generic names and adherence to Standard Treatment Guidelines (STGs), implementation is poor which requires strict monitoring and supervision by an independent agency.

However there are certain limitations to this study. Most importantly, in this paper we report the availability of medicines in the public health facilities. However, a more comprehensive assessment of the accessibility of essential medicines would also encompass issues related to selection, procurement, distribution and prescription of medicines. While, for the overall study we did undertake prescription audits, the same is not reported here. Moreover, issues related to pricing of medicines and its regulation, and market competition among the suppliers has not been considered which are recommended as potentially important research questions. Secondly, we present a description of the systems for procurement and distribution system, but the same has not been critically evaluated as it is still the early formative period of transition in terms of procurement and distribution system in both these states. Thirdly, for sake of consistency, availability was determined for a specific list of survey medicines in both the states. The medicines were part of the Essential Drug Lists (EDLs) in the two states, but do not include all medicines in the list. Nevertheless, the medicines surveyed as part of the study comprised of more than 50 % EDL medicines in both the states. Differences in quality across products was not accounted for*.* Lastly, our study did not employ WHO/Health Action International (HAI) methodology for assessing the availability of medicines which is used by several studies done in India.

We recommend similar analysis of availability of medicines in the private sector. It is also recommended to study the medicine prices across private sector providers and level of price competition in pharmaceutical market. More future research is recommended to critically analyse the various models of centralized procurement and decentralized distribution systems in various states across India, to determine the factors which improves the access to medicines.

## Conclusion

Strengthening the public sector availability of medicines is a long-term, sustainable way to reduce private expenditure on healthcare. Increased allocation of funds on medicines is of paramount importance. Robust IT systems should be used for scientific warehousing and inventory management, real-time stock monitoring and transparent centralised procurement and decentralised distribution mechanism. State governments should evaluate their procurement systems to ensure efficiencies and make necessary reforms to improve availability. The data from present study can be used as a baseline to evaluate the effectiveness and cost-effectiveness of the interventions being undertaken in Punjab and Haryana to establish robust centralised procurement and decentralised distribution systems for medicines.

## Additional files

Additional file 1:
**Table S1.** (DOCX 42 kb) Additional file 2:
**Table S2.** Availability of Medicines (%) by level of Public Health facilities in the Two States of North India (DOCX 54 kb)
