# Supplementary material for: Availability of medicines in public sector health facilities of two North Indian States
Source: BMC Pharmacol Toxicol. 2015 Dec 23;16:43. doi: 10.1186/s40360-015-0043-8 (PMC4690305; doi:10.1186/s40360-015-0043-8)
Supplement: Additional file 1: — Table S1. (DOCX 42 kb) [file 40360_2015_43_MOESM1_ESM.docx]

| ***Form No. 3*** | | | | | | | | | | |
| --- | --- | --- | --- | --- | --- | --- | --- | --- | --- | --- |
| ***(I) Facility Level Medicine Availability and Stock Out Tool for Health Facility*** | | | | | | | | | | |
| Questionnaire Number (in three digit e.g. 001) | | | | |  | | | | | |
| Team Number | | | | |  | | | | | |
| State (with State Code) (Ref. Annexure - I) | | | | |  | | | | | |
| District in Which Facility is Located  (with District Code) (Ref. Annexure –II) | | | | |  | | | | | |
| Type and name of Facility | | | | | Medical College 1  District Hospital 2  Sub-Divisional Hospital 3  CHC 4  PHC 5 | | | | | |
| Date of Interview | | | | |  | | | | | |
| Job title of respondent (Registration certificate / license details, if private facility) | | | | | Medical Officer 1  Pharmacist 2  Procurement Officer 3  Others 4 | | | | | |
| **(II) Facility Level Case Load** | | | | | | | | | | |
| **Number of Outpatients** | | **Day (Previous)** | | **Week (Previous)** | | | **Month (Previous)** | | | **Year** |
| *2012-2013* | |  | |  | | |  | | |  |
| *2011-2012* | | **Not Applicable** | | | | | | | |  |
| *2010-2011* | |  |  |  |  |  |  |  |  |  |
| **Number of Inpatients** | |  | |  | | |  | | |  |
| *2012-2013* | |  | |  | | |  | | |  |
| *2011-2012* | | **Not Applicable** | | | | | | | |  |
| *2010-2011* | |  |  |  |  |  |  |  |  |  |
| **(III) Budget Allocation/Expenditure (Rs.)** | | | | | | | | | | |
|  | **Overall Budget** | **Other State Program**  **Budget (1)** | **Other State Program Budget (2)** | | | **NRHM Budget** | | **Other National Programs** | | **Others** |
| 2012-2013 |  |  |  | | |  | |  | |  |
| 2011-2012 |  |  |  | | |  | |  | |  |
| 2010-2011 |  |  |  | | |  | |  | |  |
| 2009-2010 |  |  |  | | |  | |  | |  |
| 2008-2009 |  |  |  | | |  | |  | |  |
| **(IV) Storage Conditions** | | | | | | | | | | |
| Do you have dedicated warehouse or storage space for drugs? | | | | | | | | | Yes_________1  No__________2  Don’t know__3 | |
| Is there a method in place to control temperature (e.g. roof and ceiling with space between them in hot climates, air conditioners, fans, etc.)? | | | | | | | | | Yes_________1  No__________2  Don’t know__3 | |
| Are there windows that can be opened or there are air vents? | | | | | | | | | Yes_________1  No__________2  Don’t know__3 | |
| Is there a cold storage in the facility? | | | | | | | | | Yes_________1  No__________2  Don’t know__3 | |
| Is there a regularly filled in temperature chart for the cold storage? | | | | | | | | | Yes_________1  No__________2  Don’t know__3 | |
| Are medicines stored directly on the floor? | | | | | | | | | Yes_________1  No__________2  Don’t know__3 | |
| Are Medicines stored in a systematic way (e.g. alphabetical, pharmacological)? | | | | | | | | | Yes_________1  No__________2  Don’t know__3 | |
| Is there an evidence of pests in the area? | | | | | | | | | Yes_________1  No__________2 | |
| Is inventory management done using first-expiry-first out (FEFO) or First in first Out (FIFO)? | | | | | | | | | FEFO_______1  FIFO_______2  None______3 | |
| How often do you indent drugs for your facility-capture the response in number of days? | | | | | | | | |  | |
| What is the average number of drugs that you indent each time (number of drugs and not the type of drugs)? | | | | | | | | |  | |
| Do you receive all indented drugs? | | | | | | | | |  | |
| What is the average number of drugs received in last three indents (% of the number of drugs indented)? | | | | | | | | |  | |
| Do you always get the drugs indented or you also receive non indented drugs? | | | | | | | | | Indented only  Indented +Non Indented | |
| Which are the major drugs you indent? (collect photocopy of the indent)? | | | | | | | | |  | |
| Do you consult any one before indenting? | | | | | | | | |  | |
| If yes, whom do you consult and Why? | | | | | | | | |  | |
| Who is responsible for indenting of drugs at your facility? | | | | | | | | |  | |
| How is payment done of drugs that you receive at your facility? | | | | | | | | |  | |
| How much time does it take for you to receive indented drugs from the day of indent? | | | | | | | | |  | |
| **(V) Human Resources** | | | | | | | | |  | |
| Who manages the drug procurement system at the facility level? | | | | | | | | | Medical Officer  Pharmacist  Manager  Other-Pl. specify  (____________) | |
| Was Pharmacist there during the time of visit? | | | | | | | | | Yes_________1  No__________2  Don’t know__3 | |
| Who is dispensing drugs during the time of visit? | | | | | | | | | Pharmacist  Health Assistant  Nurse  Untrained staff  Do not know | |

| **Drug availability at Primary Level** | | | | | | |
| --- | --- | --- | --- | --- | --- | --- |
| **Drug Code** | **Drug Name** | **Type of formulation** | **Dosage** | **Availability on the day of Survey**  **(Yes/No)** | **Number of days of stock outs in last 6 months (manual check of stock register)** | **Is there expired medicine on shelf (Y/N)** |
| P01 | Acetyl salicylic acid | Tablets | 75mg, 100mg, 350 mg soluble / dispersible |  |  |  |
| P02 | Activated Charcoal | Oral |  |  |  |  |
| P03 | Adrenaline Bitartrate | Injection | 1 mg / ml |  |  |  |
| P04 | Albendazole | Suspension | 200 mg/ 5 ml |  |  |  |
| P05 | Albendazole | Tablets | 400 mg |  |  |  |
| P06 | Alprazolam | Tablets | 0.25 mg; 0.5 mg |  |  |  |
| P07 | Aluminium Hydroxide + Magnesium Hydroxide | Tablet / Suspension |  |  |  |  |
| P08 | Amlodipine | Tablets | 2.5 mg; 5 mg |  |  |  |
| P09 | Atenolol | Tablets | 50mg; 100 mg |  |  |  |
| P10 | Atropine Sulphate | Injection | 1 mg/ml |  |  |  |
| P11 | Beclomethasone Dipropionate | Inhalation | 50 µg, 250µg/dose |  |  |  |
| P12 | Benzyl benzoate | Lotion | 25 % |  |  |  |
| P13 | Betamethasone Dipropionate | Cream / Ointment | 0.05% |  |  |  |
| P14 | Calcium carbonate | Tablets | 250 mg, 500 mg |  |  |  |
| P15 | Calcium gluconate | Injection | 100mg/ml |  |  |  |
| P16 | Cetrizine | Syrup | 5 mg/ml |  |  |  |
| P17 | Cetrizine | Tablets | 10mg |  |  |  |
| P18 | Chloramphenicol | Drops/Ointment | 0.4%, 1% |  |  |  |
| P19 | Chlorpheniramine Maleate | Tablets | 4 mg |  |  |  |
| P20 | Ciprofloxacin Hydrochloride | Drops/Ointment | 0.3% |  |  |  |
| P21 | Ciprofloxacin Hydrochloride | Injection | 200 mg /100 ml |  |  |  |
| P22 | Ciprofloxacin Hydrochloride | Tablets | 250 mg, 500 mg |  |  |  |
| P23 | Co-Trimoxazole (Trimethoprim+Sulphamethoxazole) | Suspension | 160 + 800 mg; 40 + 200 mg / 5 ml |  |  |  |
| P24 | Co-Trimoxazole (Trimethoprim+Sulphamethoxazole) | Tablets | 80 + 400 mg, |  |  |  |
| P25 | Cyanocobalamin | Injection | 1 mg/ml |  |  |  |
| P26 | Dexamethasone | Injection | 4 mg / ml |  |  |  |
| P27 | Dexamethasone | Tablets | 0.5 mg |  |  |  |
| P28 | Diazepam | Injection | 5 mg / ml |  |  |  |
| P29 | Diazepam | Tablets | 5 mg |  |  |  |
| P30 | Dicyclomine Hydrochloride | Injection | 10 mg / ml |  |  |  |
| P31 | Dicyclomine Hydrochloride | Tablets | 10 mg |  |  |  |
| P32 | Domperidone | Syrup | 1 mg / ml |  |  |  |

| **Drug Code** | **Drug Name** | **Type of formulation** | **Dosage** | **Availability on the day of Survey**  **(Yes/No)** | **Number of days of stock outs in last 6 months (manual check of stock register)** | **Is there expired medicine on shelf (Y/N)** |
| --- | --- | --- | --- | --- | --- | --- |
| P33 | Domperidone | Tablets | 10 mg |  |  |  |
| P34 | Ethinylestradiol + Levonorgesterol | Tablets | 0.03 mg +0.15 mg |  |  |  |
| P35 | Ferrous Sulphate/ Fumrate | Tablets | Tablets equivalent to 60 mg elemental iron |  |  |  |
| P36 | Fluoxetine hydrochloride | Capsules | 20 mg |  |  |  |
| P37 | Folic Acid | Tablets | 1 mg , 5mg |  |  |  |
| P38 | Furosemide | Injection | 10 mg/ ml |  |  |  |
| P39 | Furosemide | Tablets | 40mg |  |  |  |
| P40 | Gentian Violet | Paint | 0.5%; 1% |  |  |  |
| P41 | Glibenclamide | Tablets | 2.5 mg; 5mg |  |  |  |
| P42 | Glyceryl Trinitrate | Injection | 5mg/ml |  |  |  |
| P43 | Glyceryl Trinitrate | Sublingual Tablets | 0.5 mg |  |  |  |
| P44 | Hydrocortisone sodium succinate | Injection | 100 mg, 200mg, 400 mg |  |  |  |
| P45 | Ibuprofen | Syrup | 100mg/5ml |  |  |  |
| P46 | Ibuprofen | Tablets | 200 mg, 400 mg |  |  |  |
| P47 | Insulin Injection (Soluble) | Injection | 40 IU / ml |  |  |  |
| P48 | Intermediate Acting (Lente/NPH Insulin) | Injection | 40 IU / ml |  |  |  |
| P49 | Ipratropium bromide | Inhalation | 20µg/metered dose |  |  |  |
| P50 | Isosorbide 5 Mononitrate/ Dinitrate | Tablets | 10 mg, 20 mg |  |  |  |
| P51 | Ketamine Hydrochloride | Injection | 10 mg / ml; 50 mg / ml |  |  |  |
| P52 | Levodopa+ Carbidopa | Tablets | 100 mg+10 mg; 250 mg +25 mg; 100 mg+25 mg |  |  |  |
| P53 | Levothyroxine | Tablets | 50µg; 100 µg |  |  |  |
| P54 | Lignocaine Hydrochloride | Injection | 1-2%, |  |  |  |
| P55 | Lignocaine Hydrochloride | Spinal | 5% +7.5% Glucose |  |  |  |
| P56 | Lignocaine Hydrochloride | Topical Forms | 2-5%, |  |  |  |
| P57 | Mannitol | Injection | 10%, 20% |  |  |  |
| P58 | Medroxy Progesterone Acetate | Tablets | 5mg; 10mg |  |  |  |
| P59 | Metformin | Tablets | 500mg |  |  |  |
| P60 | Methyl Ergometrine | Injection | 0.2mg/ml |  |  |  |
| P61 | Methyl Ergometrine | Tablets | 0.125mg |  |  |  |
| P62 | Metronidazole | Injection | 500 mg /100 ml |  |  |  |
| P63 | Metronidazole | Tablets | 200 mg, 400 mg |  |  |  |
| P64 | Multivitamins (As per Schedule V of Drugs and Cosmetics Rules) | Tablets |  |  |  |  |
| P65 | N-acetylcysteine | Injection | 200 mg/ml (5 ml) |  |  |  |

| **Drug Code** | **Drug Name** | **Type of formulation** | **Dosage** | **Availability on the day of Survey**  **(Yes/No)** | **Number of days of stock outs in last 6 months (manual check of stock register)** | **Is there expired medicine on shelf (Y/N)** |
| --- | --- | --- | --- | --- | --- | --- |
| P66 | Neomycin + Bacitracin | Ointment | 5 mg + 500 IU / g |  |  |  |
| P67 | Normal Saline | Injection | 0.9% |  |  |  |
| P68 | Omeprazole | Capsules | 10mg, 20mg, 40mg |  |  |  |
| P69 | Oral Rehydration Salts | Powder for Solution | As per IP |  |  |  |
| P70 | Paracetamol | Syrup | 125 mg / 5ml |  |  |  |
| P71 | Paracetamol | Tablets | 500 mg |  |  |  |
| P72 | Pheniramine Maleate | Injection | 22.75 mg / ml |  |  |  |
| P73 | Phenytoin Sodium | Tablets or Capsules | 50mg,100 mg |  |  |  |
| P74 | Phenytoin Sodium | Syrup | 200 mg/ml |  |  |  |
| P75 | Phenytoin Sodium | Injection | 20 mg/5ml |  |  |  |
| P76 | Polyvalent Antisnake Venom | Injection | 10 ml |  |  |  |
| P77 | Povidone Iodine | Solution or Ointment | 5% |  |  |  |
| P78 | Pralidoxime Chloride(2-PAM) | Injection | 25 mg/ml |  |  |  |
| P79 | Prednisolone | Tablets | 5mg,10mg, 20 mg |  |  |  |
| P80 | Prednisolone Acetate | Drops | 0.1% |  |  |  |
| P81 | Premix Insulin 30:70 injection | Injection | 40IU/ml |  |  |  |
| P82 | Promethazine | Syrup | 5 mg / 5 ml |  |  |  |
| P83 | Rabies Vaccine | Injection |  |  |  |  |
| P84 | Ranitidine | Injection | 25 mg / ml |  |  |  |
| P85 | Salbutamol sulphate | Inhalation | 100µg/dose |  |  |  |
| P86 | Salbutamol sulphate | Syrup | 2mg/5ml |  |  |  |
| P87 | Salbutamol sulphate | Tablets | 2mg, 4mg |  |  |  |
| P88 | Silver Sulphadiazine | Cream | 1% |  |  |  |
| P89 | Sodium Valproate | Syrup | 200 mg/ml |  |  |  |
| P90 | Sodium Valproate | Tablets | 200mg,500 mg |  |  |  |
| P91 | Tetanus Toxoid | Injection |  |  |  |  |
| P92 | Vitamin A | Tablets Capsules | 5000 IU, 50000IU, 100000 IU |  |  |  |

| **Drug Availability at Secondary level of it is inclusive of the list with codes P** | | | | | | |
| --- | --- | --- | --- | --- | --- | --- |
| **Drug Code** | **Drug Name** | **Type of formulation** | **Dosage** | **Availability on the day of Survey (Yes or No response)** | **Number of days of stock outs in last 6 months (manual check of the stock register)** | **Is there expired medicine on shelf (Y/N)** |
| S01 | Acetazolamide | Tablets | 250 mg |  |  |  |
| S02 | Acyclovir | Injection/Suspension | 400 mg / 5 ml |  |  |  |
| S03 | Acyclovir | Tablets | 200mg, 400mg, 250mg, 500mg |  |  |  |
| S04 | Amoxicillin | Capsules | 250 mg, 500 mg |  |  |  |
| S05 | Amoxicillin | Powder for suspension | 125 mg / 5 ml |  |  |  |
| S06 | Amphotericin B | Injection | 50 mg |  |  |  |
| S07 | Azithromycin | Injection | 500mg |  |  |  |
| S08 | Azithromycin | Suspension | 100mg/5ml |  |  |  |
| S09 | Azithromycin | Tablets | 100, 250,500mg |  |  |  |
| S10 | Cefotaxime | Injection | 125mg, 250mg, 500mg |  |  |  |
| S11 | Ceftriaxone | Injection | 250 mg, 1 g |  |  |  |
| S12 | Codeine phosphate | Syrup | 15mg/ 5ml |  |  |  |
| S13 | Codeine phosphate | Tablets | 10mg |  |  |  |
| S14 | Desferrioxamine mesylate | Injection | 500mg |  |  |  |
| S15 | Diazepam | Suppository | 5 mg |  |  |  |
| S16 | Diazepam | Syrup | 2mg/5ml |  |  |  |
| S17 | Digoxin | Elixir | 0.05 mg/ml |  |  |  |
| S18 | Digoxin | Injection | 0.25 mg/ml |  |  |  |
| S19 | Digoxin | Tablets | 0.25 mg |  |  |  |
| S20 | Dihydroergotamine | Tablets | 1mg |  |  |  |
| S21 | Dopamine Hydrochloride | Injection | 40 mg / ml |  |  |  |
| S22 | Factor VIII Concentrate | Injection | Dried |  |  |  |
| S23 | Fluconazole | Capsules or Tablets | 50mg, 100mg, 150mg, 200mg |  |  |  |
| S24 | Heparin Sodium | Injection | 1000 IU /ml; 5000 IU/ml |  |  |  |
| S25 | Iron Dextran | Injection | 50 mg iron/ml |  |  |  |
| S26 | Losartan Potassium | Tablets | 25 mg; 50 mg |  |  |  |
| S27 | Magnesium sulphate | Injection | 500 mg /ml |  |  |  |
| S28 | Methotrexate | Tablets | 5mg, 7.5mg, 10mg |  |  |  |
| S29 | Methyl Prednisolone | Injection | 40 mg/ ml |  |  |  |

| **Drug Code** | **Drug Name** | **Type of formulation** | **Dosage** | **Availability on the day of Survey (Yes or No response)** | **Number of days of stock outs in last 6 months (manual check of the stock register)** | **Is there expired medicine on shelf (Y/N)** |
| --- | --- | --- | --- | --- | --- | --- |
| S30 | Morphine Sulphate | Tablets | 10 mg |  |  |  |
| S31 | Nifedipine | Capsules Tablets Sustained release tablets/ capsules | 5 mg, 10mg 10mg, 20mg |  |  |  |
| S32 | Ondansetron | Injection | 2mg/ml |  |  |  |
| S33 | Ondansetron | Syrup | 2 mg/ml |  |  |  |
| S34 | Ondansetron | Tablet | 4mg, 8 mg |  |  |  |
| S35 | Oxytocin | Injection | 5 IU/ ml; 10IU/ml |  |  |  |
| S36 | Permethrin | Cream/ Lotion | 5% / 1%, 5% |  |  |  |
| S37 | Streptokinase | Injection | 750,000 IU; 15,00,000 IU |  |  |  |
| S38 | Tramadol | Capsule | 50 mg,100 mg |  |  |  |
| S39 | Tramadol | Injection | 50 mg/ml |  |  |  |
| S40 | Warfarin sodium | Tablets | 5 mg |  |  |  |

| **Drug Availability at Tertiary level of it is inclusive of the list with codes P and S** | | | | | | |
| --- | --- | --- | --- | --- | --- | --- |
| **Drug Code** | **Drug Name** | **Type of formulation** | **Dosage** | **Availability on the day of Survey (Yes or No response)** | **Number of days of stock outs in last 6 months (manual check of the stock register)** | **Is there expired medicine on shelf (Y/N)** |
| T01 | Allopurinol | Tablets | 100 mg |  |  |  |
| T02 | Alpha Interferon | Injection | 3 million IU |  |  |  |
| T03 | Amoxicillin + Clavulinic acid | Injection | 600mg, 1.2gm |  |  |  |
| T04 | Amoxicillin + Clavulinic acid | Powder for suspension | 228.5mg/5ml |  |  |  |
| T05 | Amoxicillin + Clavulinic acid | Tablets | 625 mg |  |  |  |
| T06 | Betaxolol Hydrochloride | Drops | 0.25%, 0.5% |  |  |  |
| T07 | Cefixime | Tablet | 100, 200mg |  |  |  |
| T08 | Clomiphene citrate | Tablets | 50mg, 100mg |  |  |  |
| T09 | Clopidogrel | Tablets | 75 mg |  |  |  |
| T10 | Cyclophosphamide | Injection | 500 mg |  |  |  |
| T11 | Cyclophosphamide | Tablets | 50 mg, 200mg |  |  |  |
| T12 | Cyclosporine | Capsules | 10mg, 25mg, 50mg, 100mg |  |  |  |
| T13 | Diclofenac | Injection | 25 mg / ml |  |  |  |
| T14 | Fresh frozen plasma | Injection |  |  |  |  |
| T15 | Glucagon | Injection | 1mg/ml |  |  |  |
| T16 | Imatinib | Tablets | 100 mg, 400 mg |  |  |  |
| T17 | Lithium Carbonate | Tablets | 300 mg |  |  |  |
| T18 | Methyl Cellulose | Injection | 2% |  |  |  |
| T19 | Mifepristone | Tablets | 200mg |  |  |  |
| T20 | Misoprostol | Tablets | 100μg |  |  |  |
| T21 | Morphine Sulphate | Tablets | 10 mg |  |  |  |
| T22 | Pantoprazole | Injection | 40 mg |  |  |  |
| T23 | Sodium Valproate | Injection | 100 mg/5ml |  |  |  |
| T24 | Tamoxifen Citrate | Tablets | 10 mg, 20 mg |  |  |  |
| T25 | Testosterone | Capsules | 40mg(as undecanoate) |  |  |  |
| T26 | Testosterone | Injection | 25mg/ml(as propionate) |  |  |  |
| T27 | Urokinase | Injection | 500,000 IU/ml; 10,00,000 IU/ml |  |  |  |
| T28 | Vancomycin Hydrochloride | Injection | 500 mg, 1 g |  |  |  |
