# Supplementary material for: Availability of medicines in public sector health facilities of two North Indian States
Source: BMC Pharmacol Toxicol. 2015 Dec 23;16:43. doi: 10.1186/s40360-015-0043-8 (PMC4690305; doi:10.1186/s40360-015-0043-8)
Supplement: Additional file 2: — Table S2. Availability of Medicines (%) by level of Public Health facilities in the Two States of North India (DOCX 54 kb) [file 40360_2015_43_MOESM2_ESM.docx]

**Table 2: Availability of Medicines (%) by level of Public Health facilities in the Two States of North India**

| Drug Code | Drug Name | Type of Formulation | Dosage | Availability (%) | | | | | | | | | |
| --- | --- | --- | --- | --- | --- | --- | --- | --- | --- | --- | --- | --- | --- |
|  |  |  |  | Haryana | | | | | Punjab | | | | |
|  |  |  |  | PHC | CHC | DH | MC | Total | PHC | CHC | DH | MC | Total |
| P01 | Acetyl salicylic acid | Tablets | 75mg, 100mg, 350 mg soluble / dispersible | 77 | 73 | 100 | 100 | 80 | 0 | 64 | 0 | 0 | 35 |
| P02 | Activated Charcoal | Oral |  | 0 | 9 | 0 | 0 | 3 | 0 | 0 | 50 | 100 | 35 |
| P03 | Adrenaline Bitartrate | Injection | 1 mg / ml | 50 | 55 | 50 | 100 | 53 | 0 | 55 | 0 | 17 | 35 |
| P04 | Albendazole | Suspension | 200 mg/ 5 ml | 23 | 45 | 50 | 100 | 35 | 0 | 91 | 0 | 67 | 70 |
| P05 | Albendazole | Tablets | 400 mg | 95 | 100 | 83 | 100 | 95 | 0 | 100 | 0 | 0 | 55 |
| P06 | Alprazolam | Tablets | 0.25 mg; 0.5 mg | 95 | 91 | 100 | 100 | 95 | 0 | 27 | 50 | 83 | 45 |
| P07 | Aluminium Hydroxide + Magnesium Hydroxide | Tablet / Suspension |  | 77 | 82 | 100 | 100 | 83 | 0 | 18 | 0 | 0 | 10 |
| P08 | Amlodipine | Tablets | 2.5 mg; 5 mg | 100 | 100 | 100 | 100 | 100 | 0 | 82 | 0 | 0 | 45 |
| P09 | Atenolol | Tablets | 50mg; 100 mg | 95 | 100 | 100 | 100 | 98 | 0 | 91 | 0 | 0 | 50 |
| P10 | Atropine Sulphate | Injection | 1 mg/ml | 68 | 82 | 67 | 100 | 73 | 0 | 100 | 0 | 0 | 55 |
| P11 | Beclomethasone Dipropionate | Inhalation | 50 µg, 250µg/dose | 41 | 36 | 33 | 0 | 38 | 0 | 18 | 0 | 0 | 10 |
| P12 | Benzyl benzoate | Lotion | 25% | 45 | 82 | 50 | 0 | 55 | 0 | 73 | 0 | 100 | 70 |
| P13 | Betamethasone Dipropionate | Cream / Ointment | 0.05% | 36 | 36 | 67 | 100 | 43 | 0 | 91 | 0 | 17 | 55 |
| P14 | Calcium carbonate | Tablets | 250 mg, 500 mg | 73 | 100 | 100 | 100 | 85 | 0 | 91 | 0 | 0 | 50 |
| P15 | Calcium gluconate | Injection | 100mg/ml | 23 | 18 | 67 | 0 | 28 | 100 | 36 | 0 | 0 | 25 |
| P16 | Cetrizine | Syrup | 5 mg/ml | 100 | 100 | 100 | 0 | 98 | 0 | 91 | 0 | 83 | 75 |
| P17 | Cetrizine | Tablets | 10mg | 100 | 100 | 100 | 100 | 100 | 0 | 73 | 0 | 0 | 40 |
| P18 | Chloramphenicol | Drops/Ointment | 0.4%, 1% | 50 | 36 | 67 | 0 | 48 | 0 | 0 | 0 | 0 | 0 |
| P19 | Chlorpheniramine Maleate | Tablets | 4 mg | 86 | 73 | 83 | 100 | 83 | 0 | 27 | 0 | 67 | 35 |
| P20 | Ciprofloxacin Hydrochloride | Drops/Ointment | 0.30% | 41 | 36 | 33 | 0 | 38 | 0 | 27 | 0 | 0 | 15 |
| P21 | Ciprofloxacin Hydrochloride | Injection | 200 mg /100 ml | 82 | 91 | 100 | 100 | 88 | 0 | 64 | 50 | 0 | 40 |
| P22 | Ciprofloxacin Hydrochloride | Tablets | 250 mg, 500 mg | 100 | 100 | 67 | 100 | 95 | 0 | 82 | 0 | 0 | 45 |
| P23 | Co-Trimoxazole (Trimethoprim+Sulphamethoxazole) | Suspension | 160 + 800 mg; 40 + 200 mg / 5 ml | 36 | 45 | 33 | 100 | 40 | 0 | 100 | 0 | 0 | 55 |
| P24 | Co-Trimoxazole (Trimethoprim+Sulphamethoxazole) | Tablets | 80 + 400 mg, | 82 | 82 | 33 | 0 | 73 | 0 | 27 | 0 | 0 | 15 |
| P25 | Cyanocobalamin | Injection | 1 mg/ml | 9 | 0 | 0 | 0 | 5 | 0 | 18 | 0 | 0 | 10 |
| P26 | Dexamethasone | Injection | 4 mg / ml | 64 | 82 | 67 | 100 | 70 | 0 | 91 | 0 | 0 | 50 |
| P27 | Dexamethasone | Tablets | 0.5 mg | 9 | 64 | 67 | 0 | 33 | 0 | 9 | 0 | 0 | 5 |
| P28 | Diazepam | Injection | 5 mg / ml | 50 | 73 | 83 | 100 | 63 | 4 | 82 | 44 | 48 | 43 |
| P29 | Diazepam | Tablets | 5 mg | 9 | 27 | 0 | 100 | 15 | 0 | 91 | 83 | 0 | 79 |
| P30 | Dicyclomine Hydrochloride | Injection | 10 mg / ml | 41 | 55 | 50 | 0 | 45 | 59 | 91 | 0 | 0 | 58 |
| P31 | Dicyclomine Hydrochloride | Tablets | 10 mg | 86 | 73 | 100 | 0 | 83 | 5 | 45 | 67 | 0 | 25 |
| P32 | Domperidone | Syrup | 1 mg / ml | 59 | 73 | 50 | 100 | 63 | 33 | 91 | 67 | 0 | 54 |
| P33 | Domperidone | Tablets | 10 mg | 95 | 82 | 83 | 100 | 90 | 91 | 91 | 100 | 0 | 90 |
| P34 | Ethinylestradiol + Levonorgesterol | Tablets | 0.03 mg +0.15 mg | 23 | 36 | 17 | 0 | 25 | 82 | 0 | 17 | 0 | 48 |
| P35 | Ferrous Sulphate/ Fumrate | Tablets | Tablets equivalent to 60 mg elemental iron | 91 | 82 | 83 | 100 | 88 | 18 | 91 | 0 | 0 | 35 |
| P36 | Fluoxetine hydrochloride | Capsules | 20 mg | 50 | 55 | 67 | 100 | 55 | 0 | 9 | 100 | 0 | 18 |
| P37 | Folic Acid | Tablets | 1 mg , 5mg | 86 | 91 | 100 | 100 | 90 | 95 | 91 | 100 | 0 | 93 |
| P38 | Furosemide | Injection | 10 mg/ ml | 68 | 82 | 50 | 0 | 68 | 95 | 91 | 83 | 0 | 90 |
| P39 | Furosemide | Tablets | 40mg | 68 | 91 | 100 | 100 | 80 | 86 | 82 | 33 | 0 | 75 |
| P40 | Gentian Violet | Paint | 0.5%; 1% | 32 | 82 | 0 | 0 | 40 | 23 | 36 | 83 | 0 | 35 |
| P41 | Glibenclamide | Tablets | 2.5 mg; 5mg | 59 | 82 | 67 | 0 | 65 | 100 | 82 | 100 | 0 | 93 |
| P42 | Glyceryl Trinitrate | Injection | 5mg/ml | 9 | 0 | 17 | 100 | 10 | 55 | 9 | 100 | 0 | 48 |
| P43 | Glyceryl Trinitrate | Sublingual Tablets | 0.5 mg | 14 | 9 | 0 | 0 | 10 | 91 | 9 | 17 | 0 | 55 |
| P44 | Hydrocortisone sodium succinate | Injection | 100 mg, 200mg, 400 mg | 82 | 82 | 100 | 100 | 85 | 9 | 91 | 100 | 0 | 45 |
| P45 | Ibuprofen | Syrup | 100mg/5ml | 23 | 18 | 17 | 0 | 20 | 59 | 18 | 67 | 0 | 48 |
| P46 | Ibuprofen | Tablets | 200 mg, 400 mg | 73 | 73 | 50 | 0 | 68 | 86 | 100 | 0 | 0 | 75 |
| P47 | Insulin Injection (Soluble) | Injection | 40 IU / ml | 18 | 36 | 100 | 0 | 35 | 0 | 73 | 17 | 0 | 23 |
| P48 | Intermediate Acting (Lente/NPH Insulin) | Injection | 40 IU / ml | 14 | 27 | 17 | 100 | 20 | 23 | 9 | 0 | 0 | 15 |
| P49 | Ipratropium bromide | Inhalation | 20µg/metered dose | 18 | 0 | 17 | 100 | 15 | 36 | 0 | 83 | 100 | 35 |
| P50 | Isosorbide 5 Mononitrate/ Dinitrate | Tablets | 10 mg, 20 mg | 64 | 55 | 67 | 0 | 60 | 73 | 64 | 83 | 0 | 70 |
| P51 | Ketamine Hydrochloride | Injection | 10 mg / ml; 50 mg / ml | 9 | 18 | 67 | 0 | 20 | 82 | 18 | 83 | 0 | 63 |
| P52 | Levodopa+ Carbidopa | Tablets | 100 mg+10 mg; 250 mg +25 mg; 100 mg+25 mg | 5 | 9 | 33 | 100 | 13 | 86 | 9 | 33 | 0 | 55 |
| P53 | Levothyroxine | Tablets | 50µg; 100 µg | 14 | 18 | 83 | 100 | 28 | 18 | 0 | 17 | 0 | 13 |
| P54 | Lignocaine Hydrochloride | Injection | 1-2%, | 59 | 64 | 83 | 0 | 63 | 0 | 100 | 100 | 0 | 43 |
| P55 | Lignocaine Hydrochloride | Spinal | 5% +7.5% Glucose | 0 | 0 | 33 | 100 | 8 | 82 | 18 | 17 | 0 | 53 |
| P56 | Lignocaine Hydrochloride | Topical Forms | 2-5%, | 41 | 36 | 83 | 100 | 48 | 9 | 0 | 67 | 0 | 15 |
| P57 | Mannitol | Injection | 10%, 20% | 23 | 82 | 100 | 0 | 50 | 77 | 82 | 83 | 0 | 78 |
| P58 | Medroxy Progesterone Acetate | Tablets | 5mg; 10mg | 5 | 9 | 33 | 0 | 10 | 77 | 9 | 83 | 0 | 58 |
| P59 | Metformin | Tablets | 500mg | 91 | 91 | 100 | 100 | 93 | 91 | 73 | 67 | 0 | 80 |
| P60 | Methyl Ergometrine | Injection | 0.2mg/ml | 59 | 45 | 67 | 0 | 55 | 55 | 100 | 100 | 0 | 73 |
| P61 | Methyl Ergometrine | Tablets | 0.125mg | 77 | 73 | 67 | 100 | 75 | 91 | 91 | 100 | 0 | 90 |
| P62 | Metronidazole | Injection | 500 mg /100 ml | 59 | 73 | 83 | 0 | 65 | 91 | 91 | 0 | 0 | 75 |
| P63 | Metronidazole | Tablets | 200 mg, 400 mg | 91 | 82 | 83 | 100 | 88 | 23 | 36 | 83 | 0 | 35 |
| P64 | Multivitamins (As per Schedule V of Drugs and Cosmetics Rules) | Tablets |  | 86 | 100 | 83 | 0 | 88 | 100 | 27 | 67 | 0 | 73 |
| P65 | N-acetylcysteine | Injection | 200 mg/ml (5 ml) | 5 | 0 | 0 | 0 | 3 | 14 | 9 | 100 | 0 | 25 |
| P66 | Neomycin + Bacitracin | Ointment | 5 mg + 500 IU / g | 9 | 0 | 17 | 0 | 8 | 95 | 0 | 83 | 0 | 65 |
| P67 | Normal Saline | Injection | 0.90% | 59 | 82 | 83 | 0 | 68 | 73 | 100 | 100 | 0 | 83 |
| P68 | Omeprazole | Capsules | 10mg, 20mg, 40mg | 64 | 100 | 83 | 100 | 78 | 73 | 18 | 17 | 0 | 48 |
| P69 | Oral Rehydration Salts | Powder for Solution | As per IP | 100 | 100 | 100 | 100 | 100 | 18 | 55 | 67 | 0 | 35 |
| P70 | Paracetamol | Syrup | 125 mg / 5ml | 77 | 64 | 67 | 100 | 73 | 77 | 100 | 0 | 0 | 70 |
| P71 | Paracetamol | Tablets | 500 mg | 100 | 100 | 100 | 100 | 100 | 0 | 91 | 17 | 0 | 28 |
| P72 | Pheniramine Maleate | Injection | 22.75 mg / ml | 73 | 100 | 83 | 100 | 83 | 14 | 82 | 83 | 100 | 45 |
| P73 | Phenytoin Sodium | Tablets or Capsules | 50mg,100 mg | 41 | 73 | 83 | 100 | 58 | 32 | 82 | 33 | 0 | 45 |
| P74 | Phenytoin Sodium | Syrup | 200 mg/ml | 0 | 9 | 0 | 0 | 3 | 14 | 18 | 100 | 0 | 28 |
| P75 | Phenytoin Sodium | Injection | 20 mg/5ml | 18 | 18 | 100 | 100 | 33 | 100 | 18 | 100 | 0 | 75 |
| P76 | Polyvalent Antisnake Venom | Injection | 10 ml | 55 | 73 | 67 | 100 | 63 | 27 | 27 | 17 | 0 | 25 |
| P77 | Povidone Iodine | Solution or Ointment | 5% | 95 | 91 | 83 | 100 | 93 | 5 | 91 | 17 | 0 | 30 |
| P78 | Pralidoxime Chloride(2-PAM) | Injection | 25 mg/ml | 9 | 9 | 33 | 100 | 15 | 0 | 0 | 100 | 0 | 15 |
| P79 | Prednisolone | Tablets | 5mg,10mg, 20 mg | 73 | 73 | 100 | 100 | 78 | 77 | 18 | 33 | 0 | 53 |
| P80 | Prednisolone Acetate | Drops | 0.10% | 5 | 9 | 33 | 0 | 10 | 14 | 0 | 50 | 0 | 15 |
| P81 | Premix Insulin 30:70 injection | Injection | 40IU/ml | 9 | 9 | 100 | 0 | 23 | 9 | 36 | 17 | 0 | 18 |
| P82 | Promethazine | Syrup | 5 mg / 5 ml | 36 | 55 | 50 | 100 | 45 | 0 | 82 | 100 | 100 | 40 |
| P83 | Rabies Vaccine | Injection |  | 14 | 45 | 100 | 0 | 35 | 95 | 45 | 33 | 0 | 70 |
| P84 | Ranitidine | Injection | 25 mg / ml | 73 | 100 | 100 | 100 | 85 | 0 | 100 | 17 | 0 | 30 |
| P85 | Salbutamol sulphate | Inhalation | 100µg/dose | 32 | 73 | 100 | 0 | 53 | 14 | 18 | 100 | 0 | 28 |
| P86 | Salbutamol sulphate | Syrup | 2mg/5ml | 59 | 64 | 83 | 100 | 65 | 27 | 73 | 17 | 0 | 38 |
| P87 | Salbutamol sulphate | Tablets | 2mg, 4mg | 77 | 82 | 100 | 0 | 80 | 5 | 91 | 100 | 0 | 43 |
| P88 | Silver Sulphadiazine | Cream | 1% | 32 | 18 | 50 | 0 | 30 | 68 | 82 | 83 | 0 | 73 |
| P89 | Sodium Valproate | Syrup | 200 mg/ml | 0 | 9 | 33 | 0 | 8 | 86 | 9 | 83 | 0 | 63 |
| P90 | Sodium Valproate | Tablets | 200mg,500 mg | 9 | 45 | 100 | 100 | 35 | 91 | 9 | 83 | 100 | 68 |
| P91 | Tetanus Toxoid | Injection |  | 86 | 91 | 83 | 100 | 88 | 86 | 73 | 17 | 0 | 70 |
| P92 | Vitamin A | Tablets; Capsules | 5000 IU; 50000IU, 100000 IU | 55 | 55 | 67 | 0 | 55 | 41 | 45 | 17 | 0 | 38 |
| S01 | Acetazolamide | Tablets | 250 mg | - | 0 | 17 | 100 | 11 | - | 0 | 17 | 0 | 6 |
| S02 | Acyclovir | Injection/Suspension | 400 mg / 5 ml | - | 0 | 0 | 0 | 0 | - | 0 | 17 | 0 | 6 |
| S03 | Acyclovir | Tablets | 200mg, 400mg, 250mg, 500mg | - | 27 | 83 | 0 | 44 | - | 64 | 100 | 0 | 72 |
| S04 | Amoxicillin | Capsules | 250 mg, 500 mg | - | 82 | 100 | 100 | 89 | - | 73 | 50 | 0 | 61 |
| S05 | Amoxicillin | Powder for suspension | 125 mg / 5 ml | - | 64 | 83 | 100 | 72 | - | 91 | 83 | 0 | 83 |
| S06 | Amphotericin B | Injection | 50 mg | - | 0 | 0 | 100 | 6 | - | 0 | 100 | 0 | 33 |
| S07 | Azithromycin | Injection | 500mg | - | 0 | 0 | 0 | 0 | - | 9 | 100 | 0 | 39 |
| S08 | Azithromycin | Suspension | 100mg/5ml | - | 45 | 67 | 100 | 56 | - | 9 | 100 | 0 | 39 |
| S09 | Azithromycin | Tablets | 100, 250,500mg | - | 91 | 100 | 100 | 94 | - | 82 | 100 | 0 | 83 |
| S10 | Cefotaxime | Injection | 125mg, 250mg, 500mg | - | 45 | 100 | 100 | 67 | - | 73 | 17 | 0 | 50 |
| S11 | Ceftriaxone | Injection | 250 mg, 1 g | - | 73 | 83 | 100 | 78 | - | 55 | 33 | 0 | 44 |
| S12 | Codeine phosphate | Syrup | 15mg/ 5ml | - | 0 | 17 | 0 | 6 | - | 9 | 67 | 0 | 28 |
| S13 | Codeine phosphate | Tablets | 10mg | - | 0 | 0 | 0 | 0 | - | 0 | 83 | 100 | 33 |
| S14 | Desferrioxamine mesylate | Injection | 500mg | - | 0 | 0 | 0 | 0 | - | 0 | 50 | 0 | 17 |
| S15 | Diazepam | Suppository | 5 mg | - | 0 | 0 | 0 | 0 | - | 18 | 17 | 0 | 17 |
| S16 | Diazepam | Syrup | 2mg/5ml | - | 0 | 0 | 0 | 0 | - | 0 | 17 | 0 | 6 |
| S17 | Digoxin | Elixir | 0.05 mg/ml | - | 0 | 17 | 0 | 6 | - | 0 | 33 | 0 | 11 |
| S18 | Digoxin | Injection | 0.25 mg/ml | - | 0 | 17 | 100 | 11 | - | 9 | 83 | 0 | 33 |
| S19 | Digoxin | Tablets | 0.25 mg | - | 27 | 100 | 100 | 56 | - | 9 | 67 | 100 | 33 |
| S20 | Dihydroergotamine | Tablets | 1mg | - | 0 | 0 | 0 | 0 | - | 9 | 100 | 0 | 39 |
| S21 | Dopamine Hydrochloride | Injection | 40 mg / ml | - | 45 | 67 | 0 | 50 | - | 18 | 17 | 0 | 17 |
| S22 | Factor VIII Concentrate | Injection | Dried | - | 0 | 17 | 0 | 6 | - | 0 | 100 | 0 | 33 |
| S23 | Fluconazole | Capsules or Tablets | 50mg, 100mg, 150mg, 200mg | - | 100 | 83 | 0 | 89 | - | 64 | 100 | 0 | 72 |
| S24 | Heparin Sodium | Injection | 1000 IU /ml; 5000 IU/ml | - | 9 | 50 | 100 | 28 | - | 0 | 83 | 0 | 28 |
| S25 | Iron Dextran | Injection | 50 mg iron/ml | - | 27 | 17 | 0 | 22 | - | 0 | 0 | 0 | 0 |
| S26 | Losartan Potassium | Tablets | 25 mg; 50 mg | - | 55 | 50 | 100 | 56 | - | 82 | 0 | 0 | 50 |
| S27 | Magnesium sulphate | Injection | 500 mg /ml | - | 73 | 100 | 100 | 83 | - | 18 | 100 | 0 | 44 |
| S28 | Methotrexate | Tablets | 5mg, 7.5mg, 10mg | - | 9 | 17 | 0 | 11 | - | 0 | 50 | 0 | 17 |
| S29 | Methyl Prednisolone | Injection | 40 mg/ ml | - | 0 | 0 | 100 | 6 | - | 9 | 0 | 0 | 6 |
| S30 | Morphine Sulphate | Tablets | 10 mg | - | 0 | 0 | 0 | 0 | - | 0 | 0 | 0 | 0 |
| S31 | Nifedipine | Capsules Tablets; Sustained release tablets/ capsules | 5 mg, 10mg; 10mg, 20mg | - | 36 | 67 | 100 | 50 | - | 55 | 100 | 0 | 67 |
| S32 | Ondansetron | Injection | 2mg/ml | - | 18 | 50 | 0 | 28 | - | 82 | 50 | 0 | 67 |
| S33 | Ondansetron | Syrup | 2 mg/ml | - | 45 | 67 | 0 | 50 | - | 18 | 83 | 0 | 39 |
| S34 | Ondansetron | Tablet | 4mg, 8 mg | - | 55 | 83 | 100 | 67 | - | 9 | 17 | 0 | 11 |
| S35 | Oxytocin | Injection | 5 IU/ ml; 10IU/ml | - | 82 | 83 | 100 | 83 | - | 91 | 17 | 0 | 61 |
| S36 | Permethrin | Cream/ Lotion | 5% / 1%, 5% | - | 64 | 83 | 100 | 72 | - | 9 | 17 | 0 | 11 |
| S37 | Streptokinase | Injection | 750,000 IU; 15,00,000 IU | - | 0 | 83 | 0 | 28 | - | 0 | 100 | 0 | 33 |
| S38 | Tramadol | Capsule | 50 mg,100 mg | - | 64 | 50 | 0 | 56 | - | 64 | 83 | 0 | 67 |
| S39 | Tramadol | Injection | 50 mg/ml | - | 45 | 83 | 0 | 56 | - | 64 | 100 | 0 | 72 |
| S40 | Warfarin sodium | Tablets | 5 mg | - | 0 | 0 | 0 | 0 | - | 0 | 17 | 0 | 6 |
| T01 | Allopurinol | Tablets | 100 mg | - | - | 50 | 0 | 43 | - | - | 17 | 0 | 14 |
| T02 | Alpha Interferon | Injection | 3 million IU | - | - | 0 | 0 | 0 | - | - | 17 | 0 | 14 |
| T03 | Amoxicillin + Clavulinic acid | Injection | 600mg, 1.2gm | - | - | 50 | 100 | 57 | - | - | 33 | 0 | 29 |
| T04 | Amoxicillin + Clavulinic acid | Powder for suspension | 228.5mg/5ml | - | - | 50 | 100 | 57 | - | - | 33 | 0 | 29 |
| T05 | Amoxicillin + Clavulinic acid | Tablets | 625 mg | - | - | 67 | 100 | 71 | - | - | 17 | 0 | 14 |
| T06 | Betaxolol Hydrochloride | Drops | 0.25%, 0.5% | - | - | 0 | 0 | 0 | - | - | 17 | 0 | 14 |
| T07 | Cefixime | Tablet | 100, 200mg | - | - | 67 | 100 | 71 | - | - | 17 | 0 | 14 |
| T08 | Clomiphene citrate | Tablets | 50mg, 100mg | - | - | 67 | 0 | 57 | - | - | 17 | 0 | 14 |
| T09 | Clopidogrel | Tablets | 75 mg | - | - | 83 | 100 | 86 | - | - | 83 | 0 | 71 |
| T10 | Cyclophosphamide | Injection | 500 mg | - | - | 0 | 100 | 14 | - | - | 17 | 0 | 14 |
| T11 | Cyclophosphamide | Tablets | 50 mg, 200mg | - | - | 0 | 0 | 0 | - | - | 83 | 0 | 71 |
| T12 | Cyclosporine | Capsules | 10mg, 25mg, 50mg, 100mg | - | - | 0 | 0 | 0 | - | - | 33 | 0 | 29 |
| T13 | Diclofenac | Injection | 25 mg / ml | - | - | 83 | 100 | 86 | - | - | 33 | 0 | 29 |
| T14 | Fresh frozen plasma | Injection |  | - | - | 33 | 0 | 29 | - | - | 100 | 0 | 86 |
| T15 | Glucagon | Injection | 1mg/ml | - | - | 0 | 0 | 0 | - | - | 33 | 0 | 29 |
| T16 | Imatinib | Tablets | 100 mg, 400 mg | - | - | 0 | 0 | 0 | - | - | 17 | 0 | 14 |
| T17 | Lithium Carbonate | Tablets | 300 mg | - | - | 67 | 100 | 71 | - | - | 17 | 0 | 14 |
| T18 | Methyl Cellulose | Injection | 2% | - | - | 0 | 0 | 0 | - | - | 17 | 0 | 14 |
| T19 | Mifepristone | Tablets | 200mg | - | - | 50 | 100 | 57 | - | - | 17 | 0 | 14 |
| T20 | Misoprostol | Tablets | 100μg | - | - | 67 | 100 | 71 | - | - | 100 | 0 | 86 |
| T21 | Morphine Sulphate | Tablets | 10 mg | - | - | 0 | 0 | 0 | - | - | 17 | 0 | 14 |
| T22 | Pantoprazole | Injection | 40 mg | - | - | 0 | 100 | 14 | - | - | 17 | 0 | 14 |
| T23 | Sodium Valproate | Injection | 100 mg/5ml | - | - | 0 | 0 | 0 | - | - | 83 | 0 | 71 |
| T24 | Tamoxifen Citrate | Tablets | 10 mg, 20 mg | - | - | 0 | 0 | 0 | - | - | 33 | 0 | 29 |
| T25 | Testosterone | Capsules | 40mg(as undecanoate) | - | - | 0 | 0 | 0 | - | - | 17 | 0 | 14 |
| T26 | Testosterone | Injection | 25mg/ml(as propionate) | - | - | 0 | 0 | 0 | - | - | 83 | 0 | 71 |
| T27 | Urokinase | Injection | 500,000 IU/ml; 10,00,000 IU/ml | - | - | 33 | 0 | 29 | - | - | 83 | 0 | 71 |
| T28 | Vancomycin Hydrochloride | Injection | 500 mg, 1 g | - | - | 17 | 0 | 14 | - | - | 17 | 0 | 14 |

***Note: Figures given in the table are percentages. Medicines codes starting from ‘S’ are only applicable for CHCs, DHs and MCs where, medicine codes starting from ‘T’ are only applicable for DHs and MCs. PHC= Primary Health Centre; CHC= Community Health Centre; DH= District Hospital; MC= Medical College***
